# Impact of degenerative radiographic abnormalities and vertebral fractures on spinal bone density of women with osteoporosis

**DOI:** 10.1590/S1516-31802002000100003

**Published:** 2002-01-02

**Authors:** Lúcia Costa Paiva, Silvana Filardi, Aarão Mendes Pinto, Adil Samara, João Francisco Marques

**Keywords:** Osteoporosis, Vertebral fractures, Bone mineral density, Osteophytes, Aortic calcification, Osteoporose, Fraturas vertebrais, Densidade mineral óssea, Osteófitos, Calcificação de aorta

## Abstract

**CONTEXT:**

Measurements of bone density taken by dual-energy x-ray absorptiometry are the most accurate procedure for the diagnosis of osteoporosis. This procedure has the disadvantage of measuring the density of all mineral components, including osteophytes, vascular and extra vertebral calcifications. These alterations can influence bone density results and densitometry interpretation.

**OBJECTIVE:**

To correlate radiography and densitometry findings from women with osteoporosis, analyzing the influence of degenerative processes and vertebral fractures on the evaluation of bone density.

**DESIGN:**

Retrospective study.

**SETTING:**

Osteoporosis outpatients’ clinic at Hospital das Clínicas, Universidade Estadual de Campinas.

**PARTICIPANTS:**

Ninety-six postmenopausal women presenting osteoporosis diagnosed by bone density.

**MAIN MEASUREMENTS:**

Bone mineral density of the lumbar spine and femoral neck were measured by the technique of dual-energy x-ray absorptiometry, using a LUNAR-DPX densitometer. Fractures, osteophytes and aortic calcifications were evaluated by simple x-rays of the thoracic and lumbar spine.

**RESULTS:**

The x-rays confirmed vertebral fractures in 41.6%, osteophytes in 33.3% and calcifications of the aorta in 30.2%. The prevalence of fractures and aortic calcifications increased with age. The mean bone mineral density was 0.783g/cm and the mean T-score was −3.47 DP. Neither fractures nor aortic calcifications had significant influence on bone mineral density (P = 0.36 and P = 0.09, respectively), despite the fractured vertebrae having greater bone mineral density (P < 0.02). Patients with lumbar spine osteophytes showed greater bone mineral density (P = 0.04). Osteophytosis was associated with lumbar spine bone mineral density after adjustment for fractures and aortic calcifications by multiple regression (P = 0.01).

**CONCLUSION:**

Osteophytes and lumbar spine fractures can overestimate bone density interpretation. The interpretation of densitometry results should be carried out together with the interpretation of a simple lumbar spine x-ray in elderly women.

## RESUMO

**INTRODUÇÃO:**

A quantificação da densidade óssea através da densitometria de dupla emissão de raio X é o método de maior acurácia para o diagnóstico de osteoporose. Entretanto, esse método apresenta a desvantagem de medir a densidade de todos os componentes minerais incluindo osteófitos, calcificações vasculares e extravertebrais. Essas alterações podem influenciar os resultados da densidade óssea, dificultando a interpretação densitométrica.

**OBJETIVO:**

Correlacionar os achados radiográficos e densitométricos de mulheres com osteoporose e avaliar a influência dos processos degenerativos e fraturas vertebrais na avaliação da densidade mineral óssea da coluna lombar.

**TIPO DE ESTUDO:**

Estudo retrospectivo.

**LOCAL:**

Ambulatório de Osteoporose do Hospital das Clínicas, Universidade Estadual de Campinas.

**CASUÍSTICA:**

96 mulheres na pós-menopausa com osteoporose diagnosticada pela densitometria óssea.

**MÉTODO:**

A densidade mineral óssea da coluna lombar e do fêmur foi avaliada através da técnica da densitometria de dupla emissão de raio-X, utilizando-se um densitômetro LUNAR-DPX. As fraturas vertebrais, osteófitos e calcificações de aorta foram avaliadas através de radiografias simples de coluna torácica e lombar na projeção lateral.

**RESULTADO:**

As radiografias revelaram a presença de fraturas vertebrais em 41,6%, osteófitos em 33,3% e calcificações de aorta em 30,2%. A freqüência de fraturas e calcificações da aorta aumentou com o aumento da idade. A média da densidade mineral óssea foi de 0,783 g/cm^2^ e os valores médios de T-score de −3,47 desviopadrão. A presença de fraturas e calcificações de aorta não influenciaram significativamente a densidade mineral óssea das pacientes (P = 0,36 e P = 0,09, respectivamente) embora as vértebras fraturadas apresentassem maior densidade mineral óssea (P < 0,02). As pacientes com osteófitos nas vértebras lombares apresentaram maior densidade óssea (P = 0,04). A análise de regressão múltipla mostrou que os osteófitos estiveram associados à densidade mineral óssea da coluna lombar (P = 0,01).

**CONCLUSÃO:**

Os osteófitos e as fraturas de coluna lombar podem superestimar a densidade óssea. Sugerimos que em pacientes mais idosas, a interpretação dos resultados densitométricos sejam complementados pela radiografia simples de coluna lombar para minimizar esse efeito.

## INTRODUCTION

Osteoporosis is considered to be a public health problem due to high morbidity and mortality rates caused by fractures, particularly in elderly women. In Brazil it is estimated that osteoporosis affects around 35% of women over 45 years old.^1^ Today, measurements of bone mineral density (BMD) taken by dual-energy x-ray absorptiometry (DXA) are the most accurate procedure for the diagnosis of osteoporosis.^2^ However, measurements taken by DXA are two-dimensional and when made with an anterior-posterior projection, the most used incidence, this procedure has the disadvantage of measuring the density of all the mineral components encountered in the x-ray pathway, including osteophytes and vascular and extra-vertebral calcifications. It has been shown in some studies that these alterations can influence bone mineral density results when the measurements taken are from regions with a degenerative process.^3,4^

A study on bone mineral density in men with osteophytes was carried out by Ito et al. (1993), in which the results from DXA and quantitative computerized tomography (QCT) were compared. It concluded that QCT is more precise because it provides only trabecular measurements without the addition of osteophytes.^4^ Studies made by Orwoll et al. (1990) and Masud et al. (1993) showed that men with lumbar spine osteophytes had 15% to 24% greater bone mineral density when compared with those without osteophytes.^3,5^

Due to the superposition of the aorta in relation to the lumbar spine, some studies have speculated that the presence of aortic calcifications can overrate lumbar spine bone mineral density.^3,6-8^

Vertebral fractures, particularly vertebral collapses, can also increase bone mineral density measurements of the affected vertebrae. As they are denser than the adjacent ones, they can be detected by the decrease in vertebral height or by the heterogeneity of density measured in adjacent vertebrae.^9^

The objective of this study was to correlate radiography and densitometry findings from women with osteoporosis, analyzing the influence of osteophytes, aortic calcifications and vertebral fractures, with regard to lumbar spine bone mineral density.

## METHODS

A retrospective investigation was made of 96 postmenopausal outpatients with osteoporosis who had been followed up at the osteoporosis outpatients’ clinic at Hospital das Clínicas, UNICAMP. All of them had osteoporosis that was diagnosed using dual-energy x-ray absorptiometry (DXA) in an anterior-posterior projection, according to the World Health Organization criteria.^2^ Bone mineral density was measured using a LUNAR-DPX densitometer (LUNAR Corporation, USA). The coefficient of variation was 2% for the lumbar spine.

Simple lumbar spine x-rays in lateral and anterior-posterior projections were analyzed by two investigators. In these, they would assess each vertebra with regard to osteophytes, aortic calcifications and vertebral fractures. Vertebral fractures were defined as being present when there was a reduction in vertebral height greater than 20% in comparison with the heights of the adjacent vertebrae.^10,11^

The statistical analysis was done using the mean bone mineral density in absolute values (g/cm^2^) and in relation to young-adult values (T-score). Comparisons of means were carried out using the Student *t* test. Multiple regression analysis was done to analyze the association between degenerative abnormalities, fractures and bone mineral density. In the statistical analysis the level of significance was set at p < 0.05. The statistical analysis was done using the SAS statistics package (NC, USA).

## RESULTS

The participants’ mean age was 64 years (SD = 9.4). The mean bone mineral density for the lumbar spine (L2-L4) was 0.783g/cm^2^ (SD = 0.112), and the mean T-score was – 3.47 SD (SD = 0.93).

Vertebral fractures were present in 40 patients (41.6%). Thirty-two patients (33%) presented osteophytes and 29 (30.2%) aortic calcifications. Twenty patients presented two concomitant radiographic alterations and seven patients presented three radiographic alterations.

The distribution of the radiographic alterations, based on age groups, showed a significant increase in the frequency of fractures (P = 0.019) and in aortic calcifications (P = 0.001) with increasing age. With regard to osteophytes, there was no significant increase (Table 1).

**Table 1:** Radiological abnormalities of the lumbar spine in women with osteoporosis, according to age group (n = 96)

	*Fractures*		*Osteophytes*		*Aortic calcifications*	
***Age group***	*n*	*%*	*n*	*%*	*n*	*%*
*< 60 years*	*9*	*225*	*11*	*344*	*3*	*10.3*
*60 - 69*	*15*	*37.5*	*11*	*344*	*12*	*414*
*≥70 years*	*16*	*40.0*	*10*	*31.2*	*14*	*48.3*
*TOTAL*	*40*	*100*	*32*	*100*	*29*	*100*
P *		*0.019*		*0.495*		*0.001*

P * *Fisher test.*

The comparison of mean lumbar spine bone mineral density (g/cm^2^) with regard to the presence or absence of fractures, osteophytes or aortic calcifications is shown in Table 2.

**Table 2:** Comparison of the mean lumbar spine bone mineral density (g/cm^2^) with or without fractures, osteophytes and aortic calcifications

	*BMD*	*(g/cm^2^)*	
*X-ray Abnormalities*	*Present Mean (SD)*	*Absent Mean (SD)*	* *P*
*Osteophytes*	*0.815 (0.088)*	*0766 (0.120)*	*0.04*
*Vertebral Fractures*	*0.767(0.132)*	*0790(0.104)*	*0.36*
*Aortic Calcifications*	*0753 (0.104)*	*0795 (0.115)*	*0.09*

*BMD = bone mineral density;*

* *Student's t test.*

**Figure 1:**
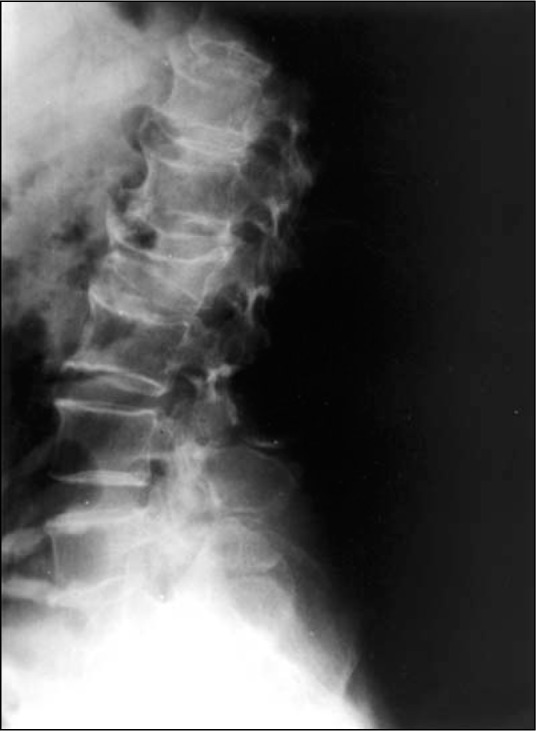
Lumbar spine x-ray showing lumbar fracture in L_2_ and multiple and large osteophytes.

**Figure 2:**
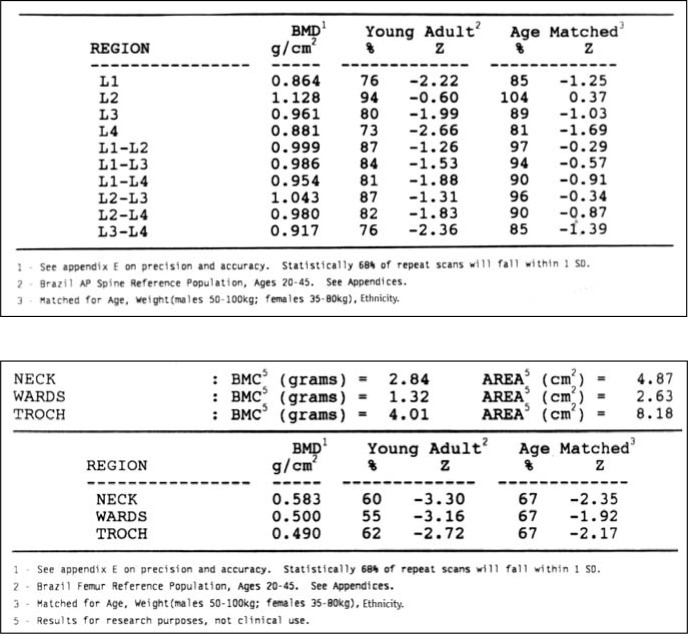
(A). Spine bone density showing an increase in BMD caused by loss of vertebral heights in L_2_ and osteophytes in L_1_-L_3_. **(B)**. Femoral bone density in the same patient showing marked osteoporosis.

Patients with osteophytes had higher mean bone mineral densities (P = 0.04). There was no significant difference in the mean bone mineral density in patients with vertebral fractures or vascular calcifications, in comparison to those with no radiological alterations (Figure 1, Figures 2A and 2B).

A later, more detailed bone mineral density analysis of each vertebra from L1 to L4 was carried out, in which they were compared with the corresponding vertebrae without fractures. This comparison of mean bone mineral density for each fractured vertebra with a non-fractured one showed an increase of bone mineral density varying from 4% to 17%. The mean bone mineral density of fractured lumbar vertebrae was significantly greater than for those with no fracture (P < 0.02) (Table 3, Figure 1, Figures 2A and 2B).

**Table 3:** Mean bone mineral density of fractured and non-fractured lumbar vertebrae

*Vertebra*	*Not Fractured*		*Fractured*		*Increase %*
	*N*	*Mean (SD)*	*N*	*Mean (SD)*	
*L_1_*	*86*	*0723 (0.112)*	*10*	*0.849(0.130)*	*17*
*L_2_*	*87*	*0.753 (0.126)*	*9*	*0789(0.195)*	*4*
*L_3_*	*94*	*0784(0.111)*	*2*	*0.879(0.019)*	*12*
*L_4_*	*94*	*0795 (0.125)*	*2*	*0.909(0.012)*	*14*
***Total***	***361***	***0.765 (0.122)***	***23***	***0.829 (0.149)***	***P < 0.02***

When potential confounders such as fractures and aortic calcifications were considered in multiple regression, an association between osteophytes and bone mineral density was revealed (P = 0.0174) (Table 4).

**Table 4:** Factors associated with lumbar spine bone mineral density by multiple regression analysis

*Independent variable*	*Coefficient*	*SE*	*t*	*P*
*Intercept*	*0779*	*0.015*	*50.212*	*0.0001*
*Osteophytes*	*0.064*	*0.026*	*2423*	*0.0174*
*Aortic calcification*	*-0.047*	*0.024*	*-1.916*	*0.0584*
*Fractures*	*-0.024*	*0.031*	*-0764*	*04466*

*r^2^ = 0.08.*

## DISCUSSION

The prevalence of alterations in our sample was high when compared with other studies and this increased with age. Dawson-Hughes & Dallal analyzed the impact of radiographic alterations with regard to loss of spinal bone mass in a group of 293 postmenopausal women with no fractures, finding a 2% prevalence for osteophytes and 11% for aortic calcifications.^12^ Another study found a 69% prevalence of osteophytes in men and women with a mean age of 69 years.^13^ These differences may be due to the diversity in the characteristics of the population studied, which was made up only of postmenopausal women presenting a densitometry diagnosis of osteoporosis, of which 40% had vertebral fractures.

The influence of osteophytes on bone mineral density has been the focus of various studies, which showed that bone mineral density was greater in vertebrae with osteophytes.^6,8,14-16^ Studies analyzing the intensity of osteophytes have shown that the greater the intensity of this abnormality, the higher the increase in bone mineral density is.^3^ It is clear that osteophytes can lead to an apparent increase in bone mineral density, although it is not clear whether this increase is associated with a decrease in fractures.

With regard to calcification of the abdominal aorta, the results from this study are similar to those presented by other investigators, who likewise did not find that this type of calcification influenced bone mineral density evaluated by densitometry.^8,17^ Nevertheless, some studies have observed that large calcifications can cause a discrete increase in bone mineral density in the affected area, although this influence is small.^3,7^ Despite the controversies, aortic calcifications seem to have a much smaller effect on vertebral bone mineral density, due probably to the fact that these vascular deposits have less mineral density.^3^

The incidence of lumbar fractures in the study population was 24%, which was similar to the 20% found by other investigators.^13^ It was observed that the prevalence of thoracic and lumbar spine fractures increased from 22.5%, in the under 60-year age group, to 40% in women over 70 years of age. Other studies have shown that the prevalence, type and number of vertebral deformities increase with age and are more prevalent in women.^18-20^ However, the actual prevalence reported depends not only on the age and sex of the population measured but also upon the method of fracture definition.^13^ The criterion used for defining vertebral fractures in this study was a reduction in vertebral height greater than 20%, in comparison with the height of adjacent vertebrae. However, many authors do not consider this criterion to be ideal, as these parameters can vary along the spine and the presence of any degenerative change can confound measurements in adjacent vertebrae, leading to a false fracture diagnosis.^13,21^

When comparing bone mineral density in patients with or without fractures it was observed that the patients with vertebral fractures had a lower mean bone mineral density than was found in the group of patients without fractures. This was to be expected, as a lower bone mineral density means a higher risk of fracture. Analyzing each one of the vertebrae (L1 to L4), comparing bone mineral density in fractured vertebrae to non-fractured ones, an increase in bone mineral density was found in the fractured ones, varying from 4% to 17%. Some authors have shown that the bone mineral density of vertebrae with old fractures has an increase of around 16%, and that differences in adjacent vertebrae that are greater than 10% should be analyzed cautiously and sometimes even eliminated from a mean bone mineral density study.^9^

These results show that degenerative processes, particularly osteophytes and fractures, can hinder the interpretation of bone densitometry of the spine and may even in some cases overestimate the measurements of bone mass in the affected areas. To reduce these effects, it is suggested that, for more elderly patients, densitometry results should be accompanied by an x-ray of the lumbar spine region, particularly in patients over 70 years of age, as these alterations are more frequent at this age.
